# The reactome pathway knowledgebase 2022

**DOI:** 10.1093/nar/gkab1028

**Published:** 2021-11-12

**Authors:** Marc Gillespie, Bijay Jassal, Ralf Stephan, Marija Milacic, Karen Rothfels, Andrea Senff-Ribeiro, Johannes Griss, Cristoffer Sevilla, Lisa Matthews, Chuqiao Gong, Chuan Deng, Thawfeek Varusai, Eliot Ragueneau, Yusra Haider, Bruce May, Veronica Shamovsky, Joel Weiser, Timothy Brunson, Nasim Sanati, Liam Beckman, Xiang Shao, Antonio Fabregat, Konstantinos Sidiropoulos, Julieth Murillo, Guilherme Viteri, Justin Cook, Solomon Shorser, Gary Bader, Emek Demir, Chris Sander, Robin Haw, Guanming Wu, Lincoln Stein, Henning Hermjakob, Peter D’Eustachio

**Affiliations:** Ontario Institute for Cancer Research, Toronto, ON M5G0A3, Canada; College of Pharmacy and Health Sciences, St. John’s University, Queens, NY11439, USA; Ontario Institute for Cancer Research, Toronto, ON M5G0A3, Canada; Ontario Institute for Cancer Research, Toronto, ON M5G0A3, Canada; Ontario Institute for Cancer Research, Toronto, ON M5G0A3, Canada; Ontario Institute for Cancer Research, Toronto, ON M5G0A3, Canada; Ontario Institute for Cancer Research, Toronto, ON M5G0A3, Canada; Universidade Federal do Paraná, Curitiba, 80060-000, Brazil; European Molecular Biology Laboratory, European Bioinformatics Institute (EMBL-EBI), Wellcome Genome Campus, Hinxton, Cambridgeshire, CB10 1SD, UK; Department of Dermatology, Medical University of Vienna, 1090 Vienna, Austria; European Molecular Biology Laboratory, European Bioinformatics Institute (EMBL-EBI), Wellcome Genome Campus, Hinxton, Cambridgeshire, CB10 1SD, UK; NYU Grossman School of Medicine, New York, NY10016, USA; European Molecular Biology Laboratory, European Bioinformatics Institute (EMBL-EBI), Wellcome Genome Campus, Hinxton, Cambridgeshire, CB10 1SD, UK; National Center for Protein Sciences Beijing, Beijing Institute of Life Omics, Beijing102206, China; Chongqing Key Laboratory on Big Data for Bio Intelligence, Chongqing University of Posts and Telecommunications, Chongqing 400065, China; European Molecular Biology Laboratory, European Bioinformatics Institute (EMBL-EBI), Wellcome Genome Campus, Hinxton, Cambridgeshire, CB10 1SD, UK; Open Targets, Wellcome Genome Campus, Hinxton, Cambridgeshire, CB10 1SD, UK; European Molecular Biology Laboratory, European Bioinformatics Institute (EMBL-EBI), Wellcome Genome Campus, Hinxton, Cambridgeshire, CB10 1SD, UK; European Molecular Biology Laboratory, European Bioinformatics Institute (EMBL-EBI), Wellcome Genome Campus, Hinxton, Cambridgeshire, CB10 1SD, UK; Ontario Institute for Cancer Research, Toronto, ON M5G0A3, Canada; NYU Grossman School of Medicine, New York, NY10016, USA; Ontario Institute for Cancer Research, Toronto, ON M5G0A3, Canada; Oregon Health and Science University, Portland, OR 97239, USA; Oregon Health and Science University, Portland, OR 97239, USA; Oregon Health and Science University, Portland, OR 97239, USA; Oregon Health and Science University, Portland, OR 97239, USA; European Molecular Biology Laboratory, European Bioinformatics Institute (EMBL-EBI), Wellcome Genome Campus, Hinxton, Cambridgeshire, CB10 1SD, UK; European Molecular Biology Laboratory, European Bioinformatics Institute (EMBL-EBI), Wellcome Genome Campus, Hinxton, Cambridgeshire, CB10 1SD, UK; Centro Internacional de Entrenamiento e Investigaciones Médicas, Cali 18 # 122-135, Colombia; European Molecular Biology Laboratory, European Bioinformatics Institute (EMBL-EBI), Wellcome Genome Campus, Hinxton, Cambridgeshire, CB10 1SD, UK; Ontario Institute for Cancer Research, Toronto, ON M5G0A3, Canada; Ontario Institute for Cancer Research, Toronto, ON M5G0A3, Canada; The Donnelly Centre, University of Toronto, Toronto, ON M5S 3E1, Canada; Oregon Health and Science University, Portland, OR 97239, USA; cBio Center at Dana-Farber Cancer Institute, Boston, MA02115, USA; Ontario Institute for Cancer Research, Toronto, ON M5G0A3, Canada; Oregon Health and Science University, Portland, OR 97239, USA; Ontario Institute for Cancer Research, Toronto, ON M5G0A3, Canada; Department of Molecular Genetics, University of Toronto, Toronto, ON M5S 1A1, Canada; European Molecular Biology Laboratory, European Bioinformatics Institute (EMBL-EBI), Wellcome Genome Campus, Hinxton, Cambridgeshire, CB10 1SD, UK; National Center for Protein Sciences Beijing, Beijing Institute of Life Omics, Beijing102206, China; NYU Grossman School of Medicine, New York, NY10016, USA

## Abstract

The Reactome Knowledgebase (https://reactome.org), an Elixir core resource, provides manually curated molecular details across a broad range of physiological and pathological biological processes in humans, including both hereditary and acquired disease processes. The processes are annotated as an ordered network of molecular transformations in a single consistent data model. Reactome thus functions both as a digital archive of manually curated human biological processes and as a tool for discovering functional relationships in data such as gene expression profiles or somatic mutation catalogs from tumor cells. Recent curation work has expanded our annotations of normal and disease-associated signaling processes and of the drugs that target them, in particular infections caused by the SARS-CoV-1 and SARS-CoV-2 coronaviruses and the host response to infection. New tools support better simultaneous analysis of high-throughput data from multiple sources and the placement of understudied (‘dark’) proteins from analyzed datasets in the context of Reactome’s manually curated pathways.

## INTRODUCTION

At the cellular level, biological processes can be represented by networks of molecular reactions that enable signal transduction, transport, DNA replication, protein synthesis and intermediary metabolism. A variety of online resources capture aspects of this information at the level of individual reactions such as Rhea (1) or at the level of interaction or reaction sequences spanning various domains of biology such as KEGG (2) or MetaCyc (3). The Reactome Knowledgebase is distinctive in focusing its manual annotation effort on a single species, *Homo sapiens*, and applying a single consistent data model across all domains of biology. Processes are systematically described in molecular detail to generate an ordered network of molecular transformations, resulting in an extended version of a classic metabolic map (4). The Reactome Knowledgebase systematically links human proteins to their molecular functions, providing a resource that is both an archive of biological process descriptions and a tool for discovering novel functional relationships in data such as gene expression studies or catalogs of somatic mutations in tumor cells.

Reactome (version 78, October 2021) has entries for 10 726 (52.5%) of the 20 442 predicted human protein-coding genes (Ensembl release 104, May 2021, http://www.ensembl.org/Homo_sapiens/Info/Annotation), involved in 13 890 reactions annotated from 34 025 literature references (Table 1). These reactions are grouped into 2546 pathways (e.g. interleukin-15 signaling, phosphatidylinositol phosphate metabolism and receptor-mediated mitophagy) collected under 28 superpathways (e.g. immune system, metabolism and autophagy) that describe normal cellular functions.

**Table 1.** Reactome content, version 70 (9/2019) (4) versus 78 (9/2021).

Data type	Release 70	Release 78	Change
Human proteins	10 867	10 726	-141^a^
Proteoforms	25 849	29 466	3617
Chemicals	1856	1940	84
Reactions	12 608	13 890	1282
Human disease proteins	308	352	44
Disease variants	1599	4603	3004
Chemical drugs	217	468	251
Protein drugs	5	39	34

^a^We have temporarily removed a group of 352 orphan olfactory GPCRs that previously were annotated as pre-associated with G-proteins because this reaction mechanism has not been demonstrated for olfactory GPCRs (6,7). Current work to annotate the epigenetic selection of individual olfactory GPCRs for expression will restore the expressed orphan olfactory GPCRs to the database (8), bringing the change in number of annotated proteins since release 70 to + 212.

A ‘Disease’ superpathway collects annotations of disease counterparts of these normal cellular processes. These disease annotations cover 4603 variant proteins and their post-translationally modified forms derived from 352 gene products and annotate 1544 disease-specific reactions tagged with 623 Disease Ontology terms (5). In addition, Reactome describes the modulating effects of 507 drugs on both normal and disease processes.

Since the last NAR update, Reactome has added 1282 new reactions, 3617 new proteoforms and 3004 disease-related genetic variants. Highlights include updated and expanded annotations of signal transduction by RHO GTPases, the molecular events in sensory perception, extended annotations of DNA repair processes and disease processes resulting from DNA repair defects, and systematic catalogs of aberrant signaling due to mutations in ALK and ERBB2 proteins and the modulating effects of mutation-specific drugs on these disease signaling processes. The number of textbook-style pathway diagrams in Reactome has risen from 91 in release 70 to 150 in release 78, the number of icons in our biomolecular icon library from 1350 to 2040.

## COVID-19: STREAMLINED CURATION OF AN EMERGING VIRAL DISEASE

In response to the emergence of SARS-CoV-2 infection in late 2019 and its subsequent pandemic spread, we have annotated the molecular processes by which SARS-CoV-2 virus replicates in human cells, how host–virus interactions can trigger pathogenic host immune responses to the virus, and how candidate repurposed drugs might modulate these processes. A key feature of this work has been the development of a protocol to streamline annotation of novel viral infections based on templates derived from well-known viral infectious processes. Here, we exploited the 82% sequence identity (9) between SARS-CoV-2 and the well-studied SARS-CoV-1 virus.

To generate comprehensive high-quality annotations expeditiously and keep them up-to-date in the face of rapidly advancing research, we proceeded in three stages. First, starting in March 2020 we curated the infection process mediated by the SARS-CoV-1 coronavirus (10). Next, we used this set of SARS-CoV-1 pathways for computational inference (11) of the corresponding SARS-CoV-2 pathways based on homology between the proteomes of the two viruses. Finally, as experimental studies of SARS-CoV-2 have emerged, we have used these results to confirm and, where necessary, revise and extend the inferred SARS-CoV-2 pathways. Working with the COVID-19 Disease Map Community (12,13), we continue to revise and extend our annotations and to integrate them with annotations generated by other members of the community to maintain a comprehensive and up-to-date description of the SARS-CoV-2 infection process (Table 2).

**Table 2.** SARS-CoV-1 and -2 entities in Reactome.

	CoV-1	CoV-2
Canonical proteins	10	10
Proteoforms	150	150
Virus complexes	79	84
Interspecies complexes	29	7
Reactions	124	128

Of the 128 reactions that comprise this process in Reactome, 116 now have associated SARS-CoV-2-specific data. Of these, 39 are reactions originally inferred from SARS-CoV-1 that are now fully supported by SARS-CoV-2 data and 10 are experimentally validated SARS-CoV-2 reactions with no SARS-CoV-1 counterpart.

## ADDING CANDIDATE DRUGS TO VIRAL INFECTION PROCESSES

**Figure 1.**
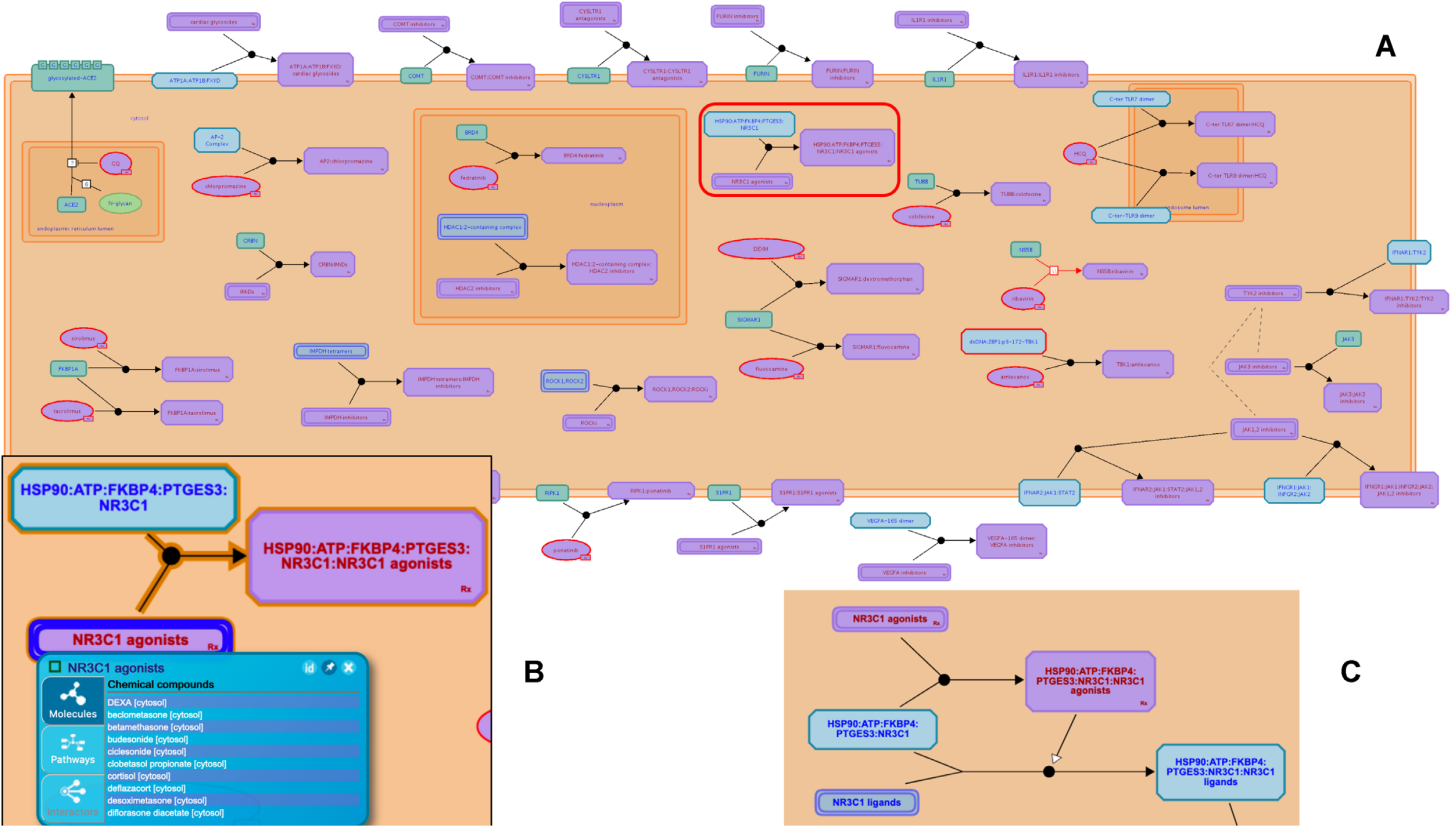
Drugs that potentially target SARS-CoV-2. (**A**) The pathway ‘Potential therapeutics for SARS’ (R-HSA-9679191, https://reactome.org/content/detail/R-HSA-9679191) is a systematic catalog of drug binding reactions; the binding of dexamethasone and other NR3C1 (glucocorticoid receptor) agonists to a complex including NR3C1 protein (R-HSA-9678925, https://reactome.org/content/detail/R-HSA-9678925), outlined in red is enlarged in (**B**), which also shows the full list of small-molecule NR3C1 agonists annotated in Reactome. The effect of this binding on the HSP90 chaperone complex (R-HSA-9690534, https://reactome.org/content/detail/R-HSA-9690534) is shown in (**C**).

We have assembled a catalog of drug molecules that could potentially be repurposed to treat COVID-19 (https://reactome.org/content/detail/R-HSA-9679191, Figure 1), incorporating the extensive drug list assembled by Gordon *et al.* (14), and supplementing it with data from recent publications. For the majority of these drugs, we have been able to incorporate ligand:target information from the Guide to Pharmacology ‘Coronavirus information’ resource (https://www.guidetopharmacology.org/GRAC/CoronavirusForward). The interaction of each drug with a viral or host cell protein target is annotated (Figure 1A,B), allowing us in many cases to incorporate the drug reactions into the SARS-CoV-2 infection pathway or host immune function pathway as negative regulators of protein functions that are annotated there (Figure 1C).

## REACTOME GENE SET ANALYSIS

**Figure 2.**
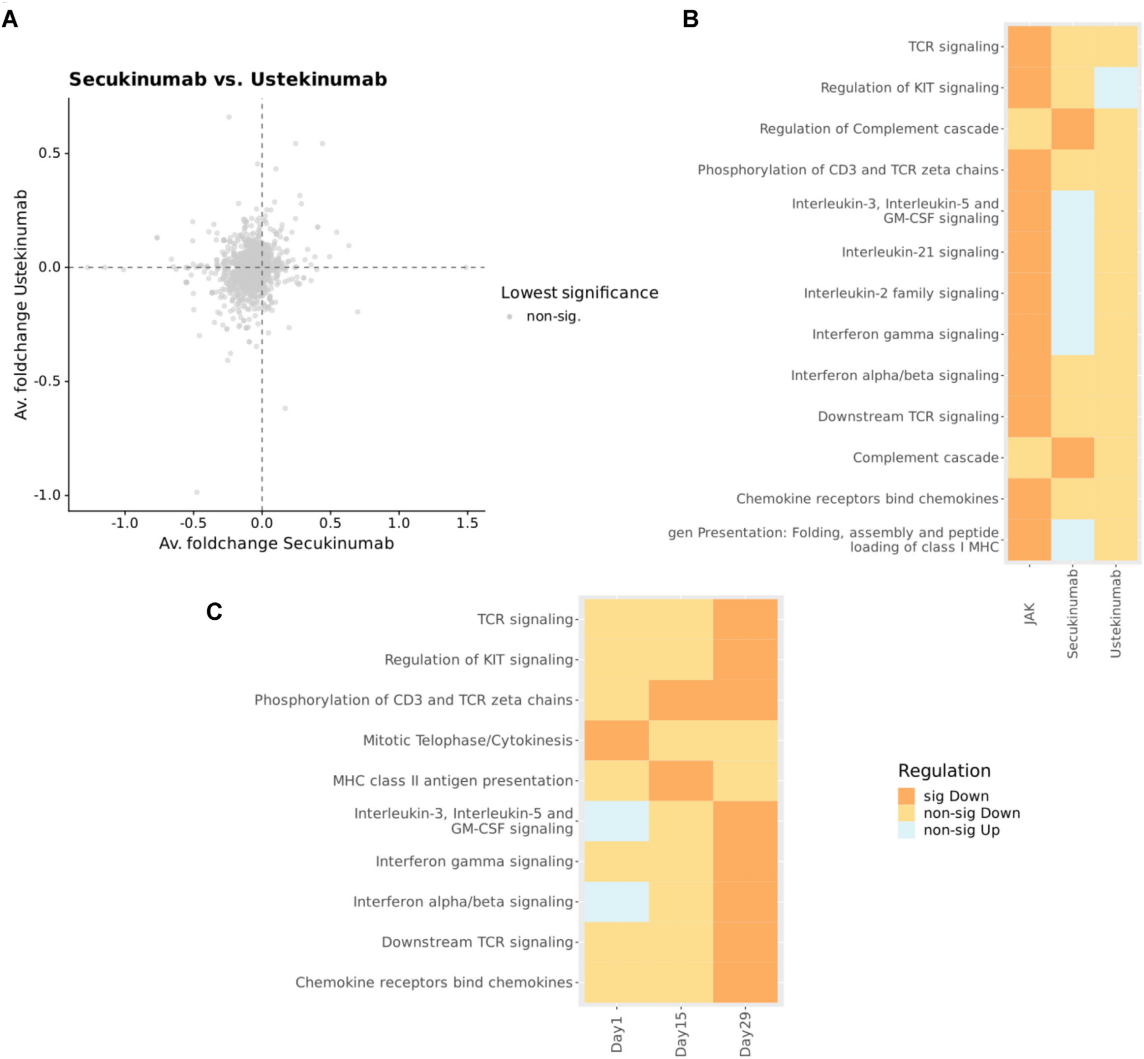
Comparative analysis of the biological effect of three compounds in atopic dermatitis using ReactomeGSA. The efficacy of Secukinumab, an IL17 inhibitor (GSE137430, transcriptomics) (17), Ustekinumab, an IL23/IL12 inhibitor (GSE140684, microarray) (18), and a JAK/SYK inhibitor (GSE133385, microarray) (19) was each compared with placebo. (**A**) Correlation analysis shows there is no shared biological effect between the drugs (example Secukinumab versus Ustekinumab). (**B**) As expected, only the JAK/SYK inhibitor led to a significant down-regulation of inflammatory pathways, consistent with the efficacy (and FDA approval) of only this class of the tested drugs in atopic dermatitis. (**C**) Comparison of different timepoints of a longitudinal study. The time series analysis of the JAK/SYK inhibitor effect shows earlier down-regulation of MHC signaling, followed by broad down-regulation of T-cell related inflammation.

The Reactome gene set analysis system (ReactomeGSA) supports comparative pathway analysis across multiple experimental datasets (15). ReactomeGSA uses gene set analysis methods that take quantitative information into consideration and performs differential expression analysis directly at the pathway level. Data from different species is automatically mapped to a common pathway space through Reactome’s internal mapping system. The gene set analysis methods are optimized for different types of ‘omics approaches including single cell RNA-sequencing (scRNA-seq) data. Public datasets can be directly integrated from ExpressionAtlas and Single Cell ExpressionAtlas (16). ReactomeGSA thereby provides easy access to multi-omics, cross-species, comparative pathway analysis to reveal key biological mechanisms by integrating large ‘omics datasets, illustrated in Figure 2. ReactomeGSA is accessible as a Reactome web-based analysis tool under the ‘Analyse gene expression’ tab at https://reactome.org/PathwayBrowser/#TOOL=AT with online documentation at https://reactome.org/userguide/analysis/gsa, as a Bioconductor R package (https://bioconductor.org/packages/release/bioc/html/ReactomeGSA.html), and programmatically using the ReactomeGSA API (https://gsa.reactome.org).

## REACTOME IDG PORTAL

While almost all the proteins encoded in the human genome are likely to have roles in normal human physiology, substantial gaps remain in catalogs of protein functions. A recent survey classified 7031 human proteins, approximately one third of the proteome, as understudied (‘dark’), with few or no published molecular annotations and not currently the subject of substantial research (20). We observed that 1940 (27.6%) of these ‘dark’ proteins were annotated components of the Reactome reaction network and an additional 890 (12.7%) were functional interactors (21), connected to the annotated network by a single hop. This motivated a collaboration with the ‘Illuminating the Druggable Genome’ (IDG) consortium to build a portal, idg.reactome.org containing a collection of web-based tools to place ‘dark’ proteins in the context of Reactome’s manually curated pathways. The portal uses data from high-throughput studies of gene expression and inferences based on sequence motifs conserved between ‘dark’ proteins and well-studied ones captured as GO biological process annotations and as protein–protein interactions. These IDG-specific tools are designed to facilitate the generation of experimentally testable hypotheses to better study the druggable genome.

**Figure 3.**
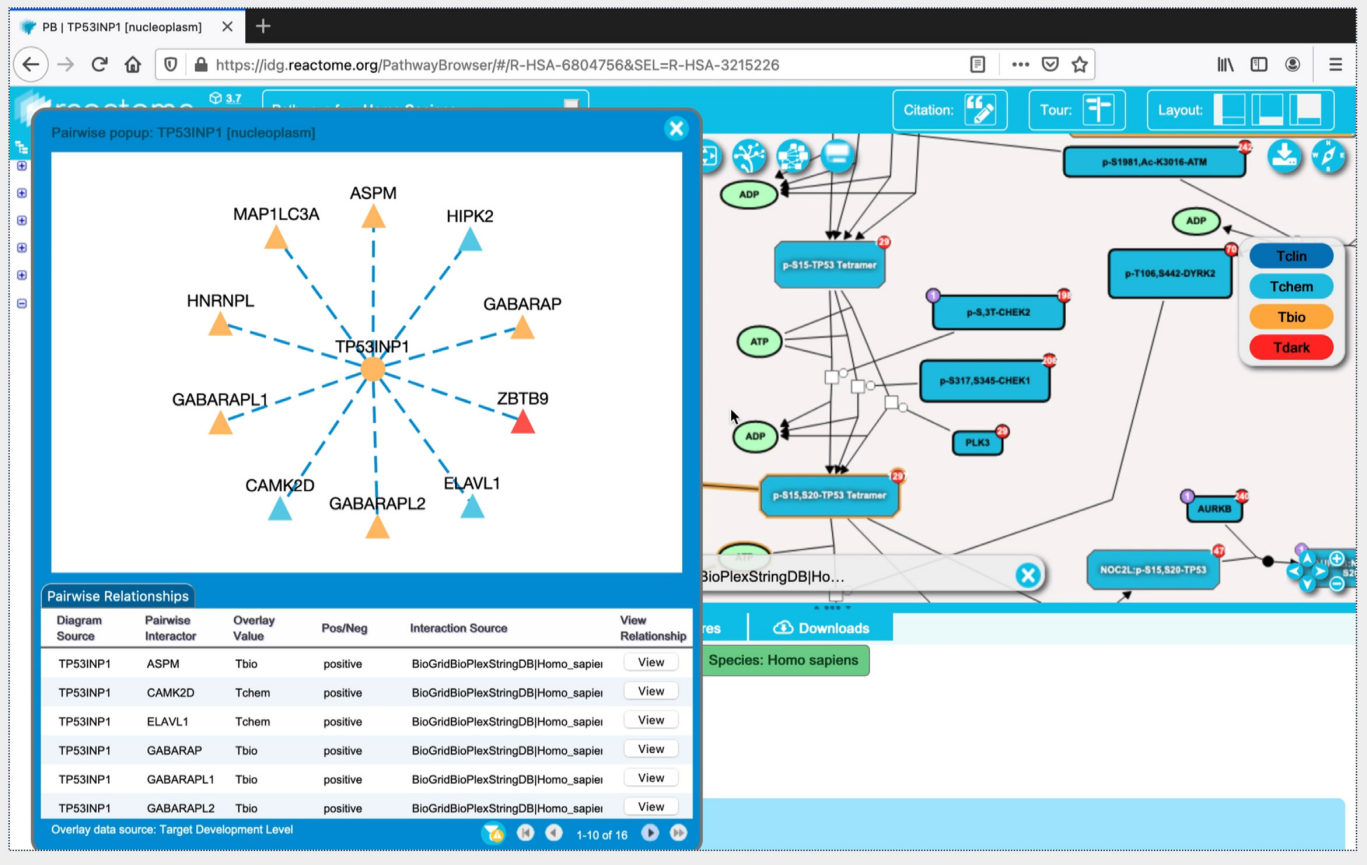
The IDG portal incorporates new features to help users visualize potential functions of understudied (‘dark’) proteins in the context of Reactome pathways. The screenshot shows some of these features, including level of knowledge of each displayed protein as a drug target (i.e. Tclin, target of drugs with known mechanism of action, Tchem, target of drugs (no mechanism), Tbio, well-characterized protein not known to be targeted by any drug, Tdark, poorly characterized protein not known to be targeted by any drug, http://juniper.health.unm.edu/tcrd/, overlaying pairwise relationships from different resources (e.g. BioGrid, BioPlex and StringDB), and a new network view.

The portal allows users to search any gene name or UniProt (22) identifier and view its placement in Reactome’s annotated pathways and in interacting pathways reachable via one-hop pairwise relationships. By default, users can view interacting pathways ranked for likely biological relevance based on functional interactions predicted from a random forest model. In order to enhance the visualization of these dark proteins, we have extended the Reactome Pathway Browser with new overlays and visualizations. In the pathway overview, users can search for a protein of interest and view its primary and interacting pathways When a pathway is opened, users are presented with an extended version of the diagram viewer, allowing them to view the knowledge levels of proteins annotated in the displayed pathway, overlay multiple tissue specific gene or protein expression values collected in the Target Central Resource Database (TCRD, http://juniper.health.unm.edu/tcrd/) at the same time, and overlay protein/protein pairwise relationships or drug/target interactions, illustrated in Figure 3. Furthermore, Reactome’s SBGN-based (https://sbgn.github.io/) pathway diagrams can be converted into functional interaction networks visualized with Cytoscape.js (https://js.cytoscape.org/). An on-line user’s guide (https://idg.reactome.org/documentation/userguide) provides instructions and solved examples illustrating the use of each of these new features. With these additional features, https://idg.reactome.org/ offers an integrative web-based platform to investigate possible functions of dark proteins and protein–drug interactions in the context of Reactome pathways.

## ACCESS TO DATA AND SOFTWARE

Reactome is open-source and open-access. All original Reactome data are available in various formats from our downloads page (https://reactome.org/download-data) and all software is available from our GitHub repository (https://github.com/reactome), under terms that allow for free reuse and redistribution.

## CONCLUSIONS

The Reactome Knowledgebase of the molecular details of human biological processes continues to grow in size and scope. Since the last NAR update, Reactome has added substantial new pathway content including coverage of the SARS-CoV-1 and SARS-CoV-2 infection processes, released ReactomeGSA, a new gene set enrichment analysis service, and created a pathway-oriented portal for the Illuminating the Druggable Genome (IDG) project.
